# Delayed Cardiovascular Response to Acute Hypotension in Heart Failure Patients Compared to Healthy Adults

**DOI:** 10.1155/ijvm/5472750

**Published:** 2026-07-11

**Authors:** Chelsea N. Cady, Soraya M. Samii, Cheryl A. Blaha, Michael J. White, Lawrence I. Sinoway, Rachel C. Drew

**Affiliations:** ^1^ Penn State Heart and Vascular Institute, Penn State College of Medicine, Hershey, Pennsylvania, USA, pennstatehershey.org; ^2^ School of Sport, Exercise and Rehabilitation Sciences, University of Birmingham, Birmingham, UK, birmingham.ac.uk; ^3^ Department of Exercise and Health Sciences, University of Massachusetts Boston, Boston, Massachusetts, USA, umb.edu

**Keywords:** acute hypotension, blood pressure, cardiovascular, heart failure, heart rate

## Abstract

Heart failure (HF) patients exhibit autonomic dysfunction. Orthostatic hypotension occurs often in HF and may be linked with altered cardiovascular responsiveness in HF. However, the cardiovascular response to acute hypotension in HF patients compared to healthy adults is unknown. Therefore, data were retrospectively analysed from eight patients with HF with reduced ejection fraction (7 male/1 female) and seven healthy adults (CON; 6 male/1 female) who underwent acute hypotension via thigh cuff deflation following 7.5 min of unilateral lower limb occlusion. Mean arterial blood pressure (MAP; photoplethysmogram) and heart rate (HR; electrocardiogram) were continuously recorded. Statistical analysis involved independent‐samples *t*‐tests. Baseline cardiovascular values were not different between groups (all *p* > 0.05). The magnitudes of the MAP peak decrease during acute hypotension and consequent HR peak increase were not different between groups (MAP: HF −11 ± 3 mmHg vs. CON −12 ± 5 mmHg, *p* = 0.756; HR: HF 3 ± 2b · min^−1^ vs. CON 4 ± 3b · min^−1^, *p* = 0.243). However, the MAP time to peak decrease and HR time to peak increase were longer in HF than CON (MAP: HF 11 ± 5 s vs. CON 6 ± 3 s, *p* = 0.041; HR: HF 10 ± 4 s vs. CON 6 ± 2 s, *p* = 0.044). HF patients exhibit a delayed cardiovascular response to acute hypotension compared to healthy adults, which may be linked with more frequent orthostatic hypotension in HF.

## 1. Introduction

Although there have been advances in the understanding of the pathophysiology and therapy of heart failure (HF), the prevalence continues to increase [1, 2]. The pathophysiology and sequelae of HF with reduced ejection fraction stem from the inability of the weakened ventricles to generate the cardiac output necessary to maintain adequate systemic perfusion [3]. There are multiple compensatory mechanisms elicited in response to this reduced perfusion, with the goal of increasing systemic vascular resistance and extracellular fluid volumes [4]. Sympathetic nervous system (SNS) activity, via the stimulation of neurons in the dorsolateral reticular formation of the medulla and transmission to preganglionic and the postganglionic fibres that synapse on the heart, blood vessels and kidneys, is augmented in HF with reduced ejection fraction [5, 6]. Although this increased SNS activation is necessary to maintain heart rate (HR), cardiac contractility and systemic vascular resistance, a deleterious consequence is the promotion of dysfunctional cardiac remodelling, which worsens cardiac function and leads to the symptoms of HF [5, 7]. Conversely, the parasympathetic nervous system (PNS) is primarily responsible for providing tonic opposition to SNS outflow, thereby decreasing HR without having a significant effect on contractility and causing vasorelaxation [8]. PNS activity is attenuated in HF, further augmenting the action of the SNS and increasing levels of norepinephrine, renin, angiotensin and vasopressin [8–10]. Diminished PNS activity in HF has been shown to cause dysregulation of HR and is related with adverse outcomes [11].

Orthostatic hypotension (OH) is characterised by a decrease in blood pressure (BP) in response to moving from lying down or sitting to standing. OH can lead to clinically significant symptoms, such as dizziness, weakness, fatigue, nausea, palpitations and syncope [12, 13]. In healthy individuals, the haemodynamic response to postural changes involves stimulation of cardiopulmonary, aortic and carotid baroreceptors, resulting in decreased PNS activity and increased SNS activity in order to augment cardiac output and systemic vascular resistance to maintain end‐organ perfusion, preventing the symptoms of OH [9, 10, 14–17]. HF patients have been shown to exhibit decreased baroreflex sensitivity, which is associated with poor survival [18]. Decreased baroreflex sensitivity due to reduced PNS activity leads to attenuated HR responses and BP that is less well controlled [19] that can result in bouts of acute hypotension. HF is commonly associated with OH [20–22], and OH can arise secondary to HF, affecting 16%–83% of hospitalised HF patients [21, 23–25]. OH has been shown to be an independent risk factor for developing HF [25–27]. Furthermore, age is one of the main nonmodifiable risk factors for developing HF [28]. Older individuals are more likely to experience OH due to age‐related autonomic dysfunction, such as reduced baroreflex sensitivity, as well as arterial and venous wall stiffening, which lead to the inability to quickly compensate for rapid changes in BP [29–33]. This inability of autonomic responses to appropriately respond to posture‐induced changes in BP can lead to frequent falls in older individuals, which is particularly problematic in this population [34]. OH also predicts the incidence of hospitalisations due to HF, independent of other risk factors [35]. The constellation of symptoms and increased incidence of hospitalisations worsens the quality of life in patients with HF [21, 25].

Some studies have investigated the immediate BP and HR responses to acute hypotension in healthy young adults [36–38] and the cardiovascular response to an orthostatic challenge in HF patients [39, 40]. Healthy adults undergoing induced acute hypotension via thigh cuff release with or without muscle metaboreflex activation have demonstrated that mean arterial BP (MAP) recovered more quickly after exercise [38]. Furthermore, sympathetically mediated vasoconstriction reduces cerebral vascular conductance in response to acute hypotension, while not contributing to overall cerebral autoregulation in healthy adults [36, 37]. In HF patients, the HR response is attenuated in response to a tilt table test [39, 40]. However, the immediate BP and HR responses to acute hypotension in HF patients compared to healthy adults have not been investigated to date. Brief bouts of hypotension (~5–10 s) can occur during typical activities of daily living, such as when moving from lying down to sitting up or from sitting to standing, and so the immediate cardiovascular response in these ~5–10 s of hypotension is very important for maintaining BP and preventing fainting. Therefore, the purpose of this study was to evaluate the cardiovascular response to acute hypotension in HF patients with reduced ejection fraction compared to healthy adults. We hypothesised that HF patients would have a larger BP decrease during acute hypotension and a smaller HR increase in response to acute hypotension compared to healthy adults. The novel findings gained from this study will have important implications for understanding the integrative physiological mechanisms underlying the increased incidence of OH in HF patients.

## 2. Materials and Methods

### 2.1. Ethics Statement

﻿This study was based on a retrospective analysis of previously collected data. The experimental protocol in which the data were previously collected was approved by the Institutional Review Board at the Penn State Milton S. Hershey Medical Center (#40359) and conformed with the Declaration of Helsinki. The study was performed in the Clinical Research Center of the Penn State Milton S. Hershey Medical Center. The purpose of the study and risks involved were explained to all participants, and written informed consent was obtained.

### 2.2. Participants

Previously collected data from eight HF patients and seven healthy adults matched for age, sex and body mass index who served as control (CON) participants were retrospectively analysed. HF patients with reduced ejection fraction were recruited from the Penn State Milton S. Hershey Medical Center Heart and Vascular Institute′s Implantable Cardiac Rhythm Device Clinic or pacemaker database. All HF patients had NYHA Class II–III HF with a mean ± standard deviation ejection fraction of 30.5*%* ± 8.9*%* (range of 17.5%–37.5%) based on echocardiographic findings from their most recent clinic visit (Table 1). Five HF patients had cardiac resynchronisation therapy defibrillators (for 543 ± 271 days prior to the study date), and three HF patients had implantable cardiac defibrillators (711 ± 730 days prior to the study date). HF patients were asked to withhold all medications (statins [*n* = 7], diuretics [*n* = 6], angiotensin‐converting enzyme inhibitors [*n* = 5], angiotensin receptor blockers [*n* = 3], antiarrhythmic [*n* = 3], antiplatelet [*n* = 3] and anticoagulant [*n* = 2] agents and diabetes medications [*n* = 1]) on the morning of the study visits except for beta‐blockers (*n* = 8), which were taken as prescribed for safety reasons. HF patients had no history of renal failure, chronic obstructive pulmonary disease or liver disease. CON participants were in good health as assessed by a history and physical exam, nonsmokers, had no history of cardiovascular disease and were not taking any medications related to cardiovascular or autonomic function. All HF patients and CON participants were normotensive.

**Table 1:** Demographic information and baseline cardiovascular measurements for the heart failure (HF) patient and healthy adult (CON) groups. Data are shown as mean ± standard deviation. NH, non‐Hispanic.

	HF	CON
Number of participants (male/female)	8 (7/1)	7 (6/1)
Age (year)	65 ± 8	65 ± 6
Race/ethnicity	8 White, NH	7 White, NH
Height (m)	1.74 ± 0.11	1.78 ± 0.08
Weight (kg)	89 ± 15	86 ± 15
Body mass index (kg·m^−1^)	29.6 ± 3.7	26.8 ± 2.9
Ejection fraction (%)	30.5 ± 8.9	—
Systolic blood pressure (mmHg)	128 ± 21	128 ± 12
Diastolic blood pressure (mmHg)	73 ± 7	82 ± 12
Mean arterial blood pressure (mmHg)	88 ± 8	97 ± 10
Heart rate (b·min^−1^)	61 ± 8	56 ± 4

### 2.3. Screening and Familiarisation

All participants attended two screening sessions prior to the experimental study. The first screening visit involved obtaining written informed consent and performing a brief history and physical exam. CON participants also performed a graded exercise stress test during this visit to rule out coronary ischemia. At the second screening visit, all participants underwent a familiarisation trial to habituate them to the experimental protocol. Data obtained during the following experimental visit were used for analysis.

### 2.4. Experimental Visit

All participants were asked to refrain from food ingestion for 8 h and abstain from caffeine and alcohol consumption and strenuous exercise performance for 24 h prior to the experimental visit. Participants were positioned in a semirecumbent position on a padded chair with their right leg flexed by 30° and their right foot strapped to a footplate, making their right lower limb parallel with the ground. A cuff was placed around the participant′s right thigh to occlude lower limb circulation when inflated. This manoeuvre was related to the original focus of the protocol in which the data were previously collected of assessing the effects of muscle mechanoreflex activation with and without concurrent muscle metaboreflex activation on baroreflex function in HF and CON (data not published). Unilateral occlusion was therefore used due to the ease of involvement of one lower limb compared to both lower limbs. Electrocardiogram patch electrodes were placed on the participant for measurement of R‐R intervals and therefore HR. A photoplethysmographic cuff was placed on one of the participant′s fingers for measurement of beat‐to‐beat BP, and a semi‐automated cuff was placed on the upper arm for BP calibration. A pneumography belt was placed around the participant′s abdomen for measurement of respiratory movement. After participants were settled for at least 10 min after instrumentation, all participants underwent a 6.5‐min resting baseline period followed by 7.5 min of unilateral lower limb circulatory occlusion via inflation of the cuff on the right thigh. After 3.5 min of cuff inflation, participants′ right calf muscle was passively stretched for 3 min, which was also related to the original focus of the protocol in which the data were previously collected but not related to the focus of the current study. One minute following the end of passive calf stretch, the thigh cuff was then deflated, which induced acute hypotension nonpharmacologically. This manoeuvre represented the stimulus related to the focus of the current study, which is the cardiovascular response to acute hypotension in HF patients compared to healthy adults. A 2‐min recovery period followed once the thigh cuff was deflated.

### 2.5. Experimental Measurements

Beat‐to‐beat MAP was measured using the photoplethysmographic finger cuff (Finometer; FMS, Arnhem, Netherlands) and calibrated to three baseline BP measurements taken using the arm cuff (SureSigns VS3; Philips, Andover, MA, United States) in offline analysis. R‐R interval was measured using a three‐lead electrocardiogram (Cardiocap/5; GE Healthcare, Waukesha, WI, United States), from which HR was derived. Respiratory movements were measured using the pneumography belt placed around the participant′s abdomen. All measurements were recorded continuously throughout the trial. ﻿An analogue‐to‐digital converter (PowerLab; ADInstruments, Castle Hill, NSW, Australia; RRID:SCR_001620) was used to sample all data at 1000 Hz. Data were recorded and displayed using LabChart software (ADInstruments, Castle Hill, NSW, Australia; RRID:SCR_023643), which was used for offline analysis.

### 2.6. Data and Statistical Analyses

Raw data files were analysed to produce beat‐to‐beat values for MAP and HR. Absolute values were calculated for the baseline period, and relative values were calculated for the change from precuff deflation during the postcuff deflation period. The exact time of cuff deflation was identified, and the MAP and HR in the beat before cuff deflation occurred were recorded to represent MAP and HR precuff deflation, respectively. MAP peak change from MAP precuff deflation, time to peak change for MAP during the induced acute hypotension, HR peak change from HR precuff deflation and time to peak change for HR in response to acute hypotension were measured. Individual data were then grouped, and data are presented as mean ± standard deviation with figures also displaying individual data. Statistical analysis involved independent‐samples *t*‐tests to compare baseline demographic and cardiovascular values, MAP peak change from MAP precuff deflation, time to peak change for MAP during acute hypotension, HR peak change from HR precuff deflation and time to peak change in HR in response to acute hypotension in HF and CON. Pearson correlation coefficients (*R*) were calculated to assess the relationships between MAP peak change from MAP precuff deflation and time to peak change for MAP during acute hypotension and HR peak change from HR precuff deflation and time to peak change in HR in response to acute hypotension in HF and CON. Statistical significance was set at *p* < 0.05, and all statistical analysis was performed using SPSS Statistics for Windows (Version 28.0; IBM, Armonk, NY, United States; RRID:SCR_016479) with figures created using Prism (Version 9.4.0 for Mac; GraphPad Software, San Diego, CA, United States; RRID:SCR_002798).

## 3. Results

### 3.1. Participant Demographics and Baseline Measurements

The demographics for the HF and CON groups are shown in Table 1. Age (*p* = 0.859), height (*p* = 0.388), weight (*p* = 0.669), body mass index (*p* = 0.137) and baseline systolic BP (*p* = 0.965), diastolic BP (*p* = 0.112), MAP (*p* = 0.062) and HR (*p* = 0.164) were not different between groups.

### 3.2. MAP During Acute Hypotension

**Figure 1:**
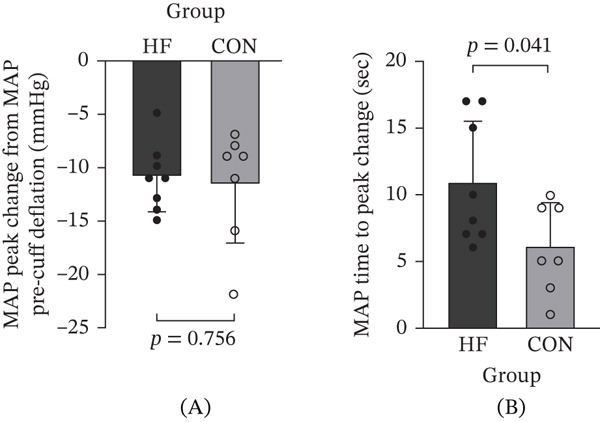
(A) Mean arterial blood pressure (MAP) peak change from MAP precuff deflation during acute hypotension in heart failure (HF) patients (*n* = 8; 7 male, 1 female) and healthy adults (CON; *n* = 7; 6 male, 1 female). (B) MAP time to peak change during acute hypotension in HF and CON. Values are individual participant data and group means ± standard deviations. Statistical analysis involved independent‐samples *t*‐tests.

Upon cuff deflation, MAP decreased in all participants, representing the induced acute hypotension, and resulted in a reflex increase in HR in both HF and CON. The magnitude of the MAP peak decrease from MAP precuff deflation during acute hypotension was similar in both groups (HF: −11 ± 3 mmHg vs. CON: −12 ± 5 mmHg; *p* = 0.756 Figure 1A). However, the MAP time to peak decrease during acute hypotension was significantly longer in HF compared to CON (HF: 11 ± 5 s vs. CON: 6 ± 3 s; *p* = 0.041; Figure 1B).

### 3.3. HR in Response to Acute Hypotension

**Figure 2:**
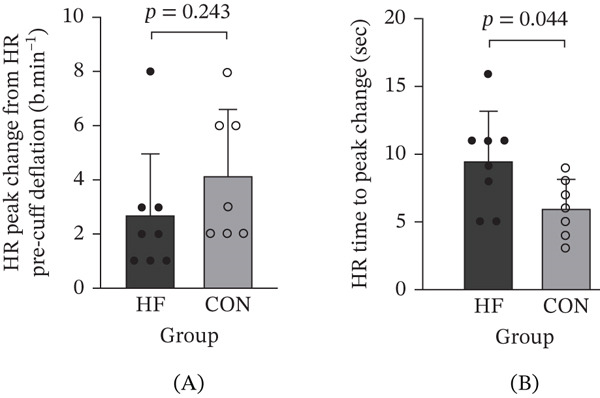
(A) Heart rate (HR) peak change from HR precuff deflation in response to acute hypotension in heart failure (HF) patients (*n* = 8; 7 male, 1 female) and healthy adults (CON; *n* = 7; 6 male, 1 female). (B) HR time to peak change in response to acute hypotension in HF and CON. Values are individual participant data and group means ± standard deviations. Statistical analysis involved independent‐samples *t*‐tests.

The magnitude of the HR peak increase from HR precuff deflation in response to acute hypotension was not different between groups (HF: 3 ± 2b · min^−1^ vs. CON: 4 ± 3b · min^−1^; *p* = 0.243; Figure 2A). However, the HR time to peak increase in response to acute hypotension was significantly longer in HF compared to CON (HF: 10 ± 4 s vs. CON: 6 ± 2 s; *p* = 0.044; Figure 2B).

### 3.4. Relationship Between Magnitude of Peak Change and Time to Peak Change for MAP and HR

**Figure 3:**
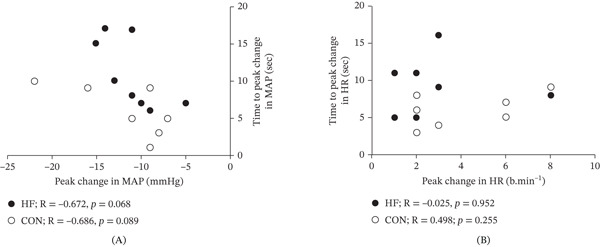
Relationship between peak change and time to peak change in (A) mean arterial blood pressure (MAP) during acute hypotension and (B) heart rate (HR) in response to acute hypotension in heart failure (HF) patients (*n* = 8; 7 male, 1 female) and healthy adults (CON; *n* = 7; 6 male, 1 female). Values are individual participant data. Statistical analysis involved Pearson correlation coefficients.

There was a strong, negative correlation between the magnitude of the MAP peak decrease from MAP precuff deflation and the MAP time to peak decrease during acute hypotension in both HF (*R* = −0.672, *p* = 0.068; Figure 3A) and CON (*R* = −0.686, *p* = 0.089; Figure 3A). However, there was a moderate positive correlation between the magnitude of the HR peak increase from HR precuff deflation and the HR time to peak increase in CON (*R* = 0.498, *p* = 0.255; Figure 3B) but no correlation in HF (*R* = −0.025, *p* = 0.952; Figure 3B).

## 4. Discussion

Findings from this study illustrate that HF patients with reduced ejection fraction exhibit a delayed cardiovascular response to acute hypotension compared to healthy adults. While the magnitudes of the MAP peak decrease during acute hypotension and HR peak increase in response to acute hypotension were similar in HF and CON, the MAP and HR time to peak changes were markedly longer in HF patients compared to CON. These findings suggest that the delayed chronotropic response of the heart to acute hypotension in HF patients with reduced ejection fraction may be an important physiological mechanism responsible for the greater occurrence of OH in this patient population.

The objective of this study was to assess the cardiovascular response to acute hypotension in HF patients compared to healthy adults. We hypothesised that HF patients with reduced ejection fraction would have a larger decrease in MAP during acute hypotension and a smaller increase in HR in response to acute hypotension compared to CON participants. Our findings demonstrate that the magnitudes of the MAP peak decrease during acute hypotension and HR peak increase in response to acute hypotension in HF were similar to CON (Figures 1A and 2A, respectively), which do not support our hypothesis. However, the intriguing findings from our study are that the MAP time to peak decrease during acute hypotension and the HR time to peak increase in response to acute hypotension were significantly longer in HF compared to CON (Figures 1B and 2B, respectively). With the HR time to peak increase in response to acute hypotension taking longer to occur in HF, the delayed ability of this acute compensatory mechanism to increase and therefore correct BP at a time of decreasing BP could have led to BP continuing to decrease and consequently decreasing over a prolonged period.

These novel findings in HF patients expand on findings from studies examining these parameters using similar techniques in healthy participants. Healthy young participants have demonstrated a decrease in MAP and reflex increase in HR in response to acute hypotension, whereas the MAP recovery is further augmented by muscle metaboreflex activation [38]. Other studies that used the approach of thigh cuff release in healthy participants showed that the SNS regulates the vasculature controlling cerebral blood flow during acute hypotension [36, 37]. When hypotension has been induced in HF patients during a tilt table test, a reflex HR increase was not seen in response to decreased right atrium and pulmonary artery filling pressures [39]. Our, and others′ [36–38], approach of deflating a standard thigh cuff to induce acute hypotension nonpharmacologically and measure resultant haemodynamic parameters avoids the use of exogenous drug interventions to examine this area, which is advantageous in a patient population such as those with HF. Using this approach, our findings advance our understanding regarding OH in HF by elucidating the chronology of the haemodynamic response to acute hypotension.

Findings from several studies have shown that HF patients have decreased baroreflex sensitivity, causing SNS activity to increase less in response to baroreceptor activation, leading to a smaller rise in BP [14, 18, 41–43]. Although we could not measure baroreflex sensitivity during the responses to acute hypotension due to their brief nature (less than ~10 s), our results are in line with the concept of reduced baroreflex sensitivity in HF patients. Attenuated baroreflex sensitivity in the HF patients in our study, caused by chronic autonomic dysfunction, could explain the slower changes in MAP and HR during acute hypotension that were observed. This likely reduced baroreflex sensitivity in these HF patients prevented an adequate haemodynamic response to acute hypotension so the clinical implications of this concept continue to be an important area in which future research should be conducted. Further, approximately 50% of HF patients experience chronotropic incompetence, the inability to adequately increase HR during physical activity, with the presence of chronotropic incompetence linked with greater mortality [44]. As autonomic dysfunction is one of the main factors contributing to chronotropic incompetence [45], future work investigating the relationship between HR responses to acute hypotension and physical activity in HF patients may be informative.

Another mechanism that could contribute to our findings is that plasma norepinephrine and renin activity are blunted in response to an orthostatic challenge in HF patients, which would prolong the associated recovery of MAP and HR [40]. Our finding that there appeared to be a strong, negative correlation between the magnitude of the MAP peak decrease and the MAP time to peak decrease during acute hypotension in HF (Figure 3A) but no correlation between the magnitude of the HR peak increase and the HR time to peak increase in response to acute hypotension in HF (Figure 3B) provides additional evidence that there is a dysregulation in the autonomic response to acute hypotension in HF patients [40]. A similar haemodynamic response to acute hypotension occurs in patients with autonomic failure due to Parkinson′s disease, perhaps implicating the importance of neurogenic causes of OH in addition to cardiovascular impairments [46]. The duration of recovery of BP during orthostasis is a clinically important factor of this reflex autonomic response because a delayed BP recovery could lead to syncope and subsequent injury from falls [47]. Importantly, the delayed increase in HR after an orthostatic challenge alone has been shown to be a predictor of mortality in healthy older individuals [48]. Future studies in which the change in important autonomic mediators, such as norepinephrine, renin and arginine vasopressin, is quantified during acute hypotension in HF may provide important information relating to the prolonged BP recovery exhibited in this population. Additionally, correlating the timing of the peak MAP and HR changes to the degree of physical symptoms experienced during acute hypotension could be beneficial in elucidating the clinical impact of OH in HF.

There are some limitations to consider for this study. A methodological limitation of this study is that HF patients did not withhold their beta‐blocker medication during their involvement. However, this approach was necessary for safety and ethical reasons for the HF patients to maintain their prescribed beta‐blocker regimen. These data from HF patients on a beta‐blocker regimen can also provide insight into responses that occur in real‐world settings. Patients with HF with reduced ejection fraction were studied so further studies are needed in patients with HF with preserved ejection fraction to assess the cardiovascular response to acute hypotension in this population of HF patients. Also, the 3‐min passive calf stretch that occurred during the protocol, which was related to the original focus of the protocol in which the data were previously collected, preceded the thigh cuff deflation that induced acute hypotension, which was the focus of the current study. However, a 1‐min period separated the end of passive calf stretch and the thigh cuff deflation that induced acute hypotension. Given the rapid nature of the reflex activation and deactivation of the muscle mechanoreflex caused by passive muscle stretch, any effects of passive calf stretch should have dissipated before acute hypotension was induced. Additionally, as participants in both the HF and CON groups identified predominantly as male, White and non‐Hispanic adults, these data should not be extrapolated to other groups such as female adults and those who identify as different racial or ethnic groups. Future studies involving different populations are warranted in this clinically important area of integrative physiology.

## 5. Conclusions

Findings from this study illustrate that HF patients exhibit a delayed cardiovascular response to acute hypotension compared to healthy adults. These novel findings provide important insight into the cardiovascular response exhibited in HF patients when experiencing acute hypotension. OH causes high morbidity and mortality, particularly in the older population [49]. HF patients, among the more clinically frail in the older population, are especially susceptible to OH because they exhibit chronic autonomic dysfunction [21]. Our findings further our understanding of the integrative physiological mechanisms involved in orthostatic intolerance in HF patients.

## Author Contributions

M.J.W. and R.C.D. conceived and designed the research. C.N.C. and R.C.D. analysed the data, and all authors interpreted the results of the analysis. C.N.C. and R.C.D. drafted the manuscript, all authors revised the manuscript and all authors approved the final version of the manuscript. All authors agree to be accountable for all aspects of the manuscript in ensuring questions related to the accuracy or integrity of any part of the manuscript are appropriately investigated and resolved.

## Funding

This study was supported by the National Institutes of Health, 10.13039/100000002, P01 HL096570 and UL TR000127 and the British Heart Foundation, 10.13039/501100000274, FS/12/18/29522.

## Conflicts of Interest

The authors declare no conflicts of interest.

## Data Availability

The de‐identified, numerical datasets used and/or analysed for this study are available from the corresponding author upon reasonable request.
